# Heterologous expression of AHL lactonase AiiK by *Lactobacillus casei* MCJΔ1 with great quorum quenching ability against *Aeromonas hydrophila* AH-1 and AH-4

**DOI:** 10.1186/s12934-020-01448-4

**Published:** 2020-10-07

**Authors:** Weiwei Dong, Yuyuan Cai, Zhilong Xu, Biao Fu, Qitong Chen, Yuxin Cui, Zhiyong Ruan, Yunxiang Liang, Nan Peng, Shumiao Zhao

**Affiliations:** 1 State Key Laboratory of Agricultural Microbiology and College of Life Science and Technology, Huazhong Agricultural University, Wuhan, 430070 China; 2 Institute of Agricultural Resources and Regional Planning, CAAS, Beijing, 100081 China

**Keywords:** Quorum sensing, Quorum quenching, AHL lactonase AiiK, *Lactobacillus casei* MCJΔ1, *Aeromonas hydrophila*

## Abstract

**Background:**

Nowadays, microbial infections have caused increasing economic losses in aquaculture industry and deteriorated worldwide environments. Many of these infections are caused by opportunistic pathogens through cell-density mediated quorum sensing (QS). The disruption of QS, known as quorum quenching (QQ), is an effective and promising way to prevent and control pathogens, driving it be the potential bio-control agents. In our previous studies, AHL lactonase AiiK was identified with many characteristics, and constitutive expression vector pELX1 was constructed to express heterologous proteins in *Lactobacillus casei* MCJΔ1 (*L. casei* MCJΔ1). In this study, recombinant strain pELCW-*aiiK*/*L. casei* MCJΔ1 (*Lc*AiiK) and wild-type *Aeromonas hydrophila* (*A. hydrophila*) were co-cultured to test the QQ ability of *Lc*AiiK against *A. hydrophila*.

**Results:**

A cell wall-associated expression vector pELCW for *L. casei* MCJΔ1 was constructed. Localization assays revealed that the expressed AiiK was anchored at the surface layer of *Lc*AiiK via vector pELCW-*aiiK*. *Lc*AiiK (OD_600_ = 0.5) degraded 24.13 μM of C_6_-HSL at 2 h, 40.99 μM of C_6_-HSL at 12 h, and 46.63 μM of C_6_-HSL at 24 h. Over 50% *Lc*AiiK cells maintained the pELCW-*aiiK* plasmid after 15 generations of cultivation without erythromycin. Furthermore, *Lc*AiiK inhibited the swimming motility, extracellular proteolytic activity, haemolytic activity and biofilm formation of *A. hydrophila* AH-1 and AH-4.

**Conclusion:**

The AHL lactonase AiiK is firstly and constitutively expressed at the surface layer of *L. casei* MCJΔ1. *Lc*AiiK displayed considerable AHL lactonase activity and great QQ abilities against *A. hydrophila* AH-1 and AH-4 by attenuating their QS processes instead of killing them. Therefore, the *Lc*AiiK can be exploited as an anti-pathogenic drug or a bio-control agent to control the AHL-mediated QS of pathogenic bacteria.

## Introduction

Microbial infections have caused increasing economic losses in aquaculture industry and deteriorated worldwide environments year by year [1]. The acute hemorrhagic septicemia in fish and diarrhea in human caused by microbial infections are reported frequently [2–5]. These symptoms are mainly caused by a kind of gram-negative opportunistic pathogens [3–5]. *Aeromonas hydrophila* (*A. hydrophila*) is a representative gram-negative opportunistic pathogen, ubiquitous in fresh and estuarine water, and can infect fish, crabs, shrimps, and even humans [4, 6]. *A. hydrophila* can also cause various symptoms including tissue swelling, necrosis, and ulceration in fish [7]. The bacterial infections in fish and human depend on the cell-density mediated system termed quorum sensing (QS) [6].

QS, a cell-to-cell communication mechanism in bacteria, coordinates the expression of specialized structural gene sets via specific receptors-sensing signal molecules when bacteria present at high cell densities [8, 9]. The *N*-Acyl homoserine lactone (AHL), a common and important QS signal molecule in gram-negative bacteria, consists of a homoserine lactone and an acyl side chain of four or more carbon atoms. AHL-mediated QS traits encompass virulence factor production, swarming motility, biofilm maturation, and so on, which impart a significant advantage in survival of bacterial populations [10–12].

AHL-mediated QS triggers the expression of potential virulence and pathogenicity factors, including production of cytotoxic enterotoxin, exoprotease, lipase and hemolysin, shifting of swarming motility, and formation of biofilm in *A. hydrophila* [6, 13]. The biofilm formation of *A. hydrophila* stimulates strong resistance to multiple antibiotics [14]. Furthermore, virulence factors and swarming motility associated with QS make the diseases and infections caused by *A. hydrophila* hard to be cured [14]. However, finding new and effective antibiotics is harder, and thus therapies for controlling the QS-mediated pathogenicity without causing the emergence of resistance are promising alternatives [15, 16]. The disruption of QS, known as quorum quenching (QQ), is of great potential in alleviating the detrimental symptoms caused by QS-mediated pathogenic events, so it could be applied as a bio-control measure for pathogenic bacterial prevention and control [9, 17–20].

In recent years, four QQ approaches have been employed to inactivate the signal molecules and alleviate the symptoms of bacterial infections caused by QS: (1) using the purified QQ enzymes, (2) expressing QQ enzymes in pathogenic bacteria, (3) isolating and identifying new QQ strains, and 4) constructing recombinant QQ strains (Table 1). In the first case, AHL lactonases AidP [9], MomL [17], AiiK [18], RmmL [19], AiiA_QSI-1_ [20], and AiiA_B546_ [21] were expressed by *Escherichia coli* or *Pichia pastoris*, purified and used to ease the pathogenicity of different pathogens. The purified QQ enzymes functioned very well; however, the purification was complicated and the cost for purification was high. The purified AHL lactonases had poor resistance to environment when applied in a real situation. In the second case, AHL lactonases AiiA [22], AiiM [23], AttM [24], and HqiA [25] were expressed in different pathogens, and their pathogenicity decreased. This approach occurred only under ideal research conditions, because pathogens are difficult to be modified in true situation. In the third case, *Bacillus licheniformis* T-1 isolated from freshwater was found to have reduced the pathogenicity of *A. hydrophila* cb15 [7]. This was due to the presence of QQ enzyme gene in the isolated *B. licheniformis* T-1; however, it is very difficult to isolate a strain with great AHL lactonase activity because of lacking suitable and efficient screening methods. In the last case, Zhang et al. constructed a recombinant QQ strain *Bb*MomL, and it significantly reduced the secretion of pathogenic factors and the pathogenicity of *P. carotovorum* subsp. carotovorum and *Pseudomonas aeruginosa* PAO1 [26]. After the recombinant QQ strain was constructed, it was applied directly against pathogens without further purification steps. Although most previous studies have focused on applying purified QQ enzymes or expressing QQ enzymes in pathogenic bacteria to inactivate the AHLs, little work has been done on constructing a recombinant QQ strain expressing the AHL lactonase on its surface to directly attenuate the symptoms caused by QS.

**Table 1:** Four QQ approaches were used to alleviate the effects of QS against pathogenic bacteria

Approaches of QQ		Enzyme type	Origin	Expression strains	Application and references
Purified QQ enzymes	AidP	AHL lactonase	*Planococcus* sp.	*E. coli* BL21	AidP attenuated the pathogenicity of *P. carotovorum* in Chinese cabbage [2]
	MomL	AHL lactonase	*M. olearia* Th120	*E. coli* BL21(DE3)	MomL attenuated virulence of *P. aeruginosa* in a *Caenorhabditis elegans* infection mode [17]
	AiiK	AHL lactonase	*K. huakuii*	*E. coli* BL21(DE3)	AiiK inhibited biofilm formation and attenuated extracellular proteolytic activity and pyocyanin production of *P. aeruginosa* PAO1 [18]
	RmmL	AHL lactonase	*R. mobilis* YJ3	*E. coli* BL21(DE3)	RmmL reduced pyocyanin production of *P. aeruginosa* PAO1 and extracellular protease activity of *V. anguillarum* VIB72 [19]
	AiiA_QSI-1_	AHL lactonase	*Bacillus* sp. QSI-1	*E. coli* BL21(DE3)	AiiA_QSI-1_ inhibited swimming motility, extracellular protease, hemolysin factor, and biofilm formation of *A. hydrophila* YJ-1 [20]
	AiiA_B546_	AHL lactonase	*Bacillus* sp. B546	*P. pastoris*	AiiA_B546_ decreased mortality rate and delayed mortality time of fish when co-injection with *A. hydrophila* in common carp [21]
Expressed QQ enzymes in pathogenic bacteria	AiiA	AHL lactonase	*Bacillus* sp. 240B1	*E. carotovora* stain SCG1	The introduction of *aiiA* gene in *E. carotovora* stain SCG1 decreased extracellular pectolytic activities and pathogenicity of *E. carotovora* [22]
	AiiM	AHL lactonase	*M. testaceum* StLB037	*P. c. c.* NBRC 3830	The introduction of *aiiM* gene in *P. carotovorum* subsp. *carotovorum* NBRC 3830 attenuated soft rot symptoms on potato slices [23]
	AttM	AHL lactonase	*A. tumefaciens* c58	*S. scabies*	The introduction of *attM* gene suppressed pathogenicity of *S. scabies* towards potato tuber [24]
	HqiA	AHL lactonase	Metagenomic library from soil	*P. c. c.* CECT 225^T^	The introduction of *hqiA* gene in plant pathogen *P. carotovorum* efficiently interfered swarming motility and maceration enzymes production [25]
Isolated and identified new QQ strains	*B. licheniformis* T-1	AHL lactonase	Freshwater culture pond sediment	No	*B. licheniformis* T-1 reduced pathogenicity of *A. hydrophila* cb15 in zebrafish coinjection [12]
Constructed recombinant QQ strains	*Bb*MomL	AHL lactonase	*M. olearia* Th120	*B. brevis*	*Bb*MomL reduced secretion of pathogenic factors and pathogenicity of *P. carotovorum* subsp. *carotovorum* and *P. aeruginosa* PAO1 [26]
	*Lc*AiiK	AHL lactonase	*K. huakuii*	*L. casei* MCJΔ1	*Lc*AiiK attenuated swimming motility, virulence factor production, and biofilm formation of *A. hydrophila* AH-1 and AH-4 (This study)

In our previous studies, a constitutive expression vector pELX1 was constructed and used to intracellularly express heterologous proteins in *Lactobacillus casei* MCJΔ1 (*L. casei* MCJΔ1) [27]. AiiK, identified as an AHL lactonase from *Kurthia huakuii* LAM0618^T^ (*K. huakuii* LAM0618^T^), showed characteristics of efficient degradation of AHLs, variable substrate spectrum, suitable thermostability, and great protease-resistance [18]. In the present study, in order to express the AiiK at surface layer of *L. casei* MCJΔ1, plasmid pELCW-*aiiK* was constructed and transformed into *L. casei* MCJΔ1. The recombinant strain pELCW-*aiiK*/*L. casei* MCJΔ1 (*Lc*AiiK) was co-cultured separately with *A. hydrophila* AH-1 and AH-4 to test its QQ ability against *A. hydrophila*, an opportunistic pathogen isolated from dead grass carp. *Lc*AiiK attenuated the production of virulence factors and inhibited the swimming activity and biofilm formation of *A. hydrophila*.

## Materials and methods

### Bacterial strains and growth conditions

Strain *K. huakuii* LAM0618^T^ was cultured in tryptic soy broth (TSB) at 30 °C with shaking. All *E. coli* strains were propagated in Luria–Bertani (LB) medium at 37 °C and 180 rpm. Strain *L. casei* MCJΔ1 was fostered in Man-Rogosa-Sharpe (MRS, tryptone 10.0 g/L, yeast extract 4.0 g/L, glucose 20.0 g/L, beef extract 8.0 g/L, NaAc·3H_2_O 8.3 g/L, Tween-80 1.0 mL/L, triammonium citrate 2.0 g/L, K_2_HPO_4_·3H_2_O 2.62 g/L, MgSO_4_·7H_2_O 0.41 g/L, MnSO_4_·H_2_O 0.056 g/L, pH 6.8) broth at 37 °C. Divalent metal ions-free MRS (DMIF-MRS, tryptone 10.0 g/L, yeast extract 4.0 g/L, glucose 20.0 g/L, beef extract 8.0 g/L, NaAc·3H_2_O 8.3 g/L, Tween-80 1.0 mL/L, triammonium citrate 2.0 g/L, K_2_HPO_4_·3H_2_O 2.62 g/L, pH 6.8) was used to test the effects of divalent metal ions on AHL lactonase activity of *Lc*AiiK. A reporter strain *Chromobacterium violaceum* CV026 was grown in LB medium at 30 °C and 180 rpm. *A. hydrophila* AH-1 and *A. hydrophila* AH-4 (16S rDNA sequences showed in supplementary material), isolated from dead grass carp (*Ctenopharyngodon idellus*), were proliferated in nutrient broth (NB, peptone 10.0 g/L, beef extract powder 3.0 g/L, and NaCl 5.0 g/L) at 30 °C and 180 rpm. Antibiotics were added to the medium when required: ampicillin (100 μg/mL) for *E. coli*, kanamycin (50 μg/mL) for *C. violaceum* CV026, and erythromycin (50 μg/mL) for recombinant *L. casei* MCJΔ1. Strains and plasmids used in this study are listed in Table 2.

**Table 2:** Bacterial strains and plasmids

Strain or plasmid	Description	Reference or source
Strains		
*Kurthia huakuii* LAM0618^T^	Wild type	ACCC 06121
*Escherichia coli* DH5α	λ^−^ф80d*lac*ZΔM15 Δ (*lacZYA*-*argF*) *U169 recA1 endA hsdR17* (r_K_^−^ m_K_^−^) *supE44 thi*-*1 gyrA relA1*	Tiangen
*Lactobacillus casei* MCJΔ1	pMC11-cured strain	Chen et al. (2014) [27]
*Chromobacterium violaceum* CV026	ATCC 31532 derivative, cviI::Tn5xylE Kmr, Smr	From Dr. Guishan Zhang
*Aeromonas hydrophila* AH-1	Wild type	Isolated from dead grass carp
*Aeromonas hydrophila* AH-4	Wild type	Isolated from dead grass carp
Plasmids		
pELX1	Expression vector, Amp^r^	Chen et al. (2014) [27]
pUC55-*NlpC*	pUC55 containing *NlpC* gene	Constructed by BGI
pELCW	Expression vector, Amp^r^	This study
pELCW-*aiiK*	pELCW containing *aiiK* gene	This study

### Construction of expression vectors pELCW and pELCW-*aiiK*

The expression vector pELCW was constructed based on pELX1 [27]. The *NlpC* gene encoding a cell wall-associated protein (accession: WP_022667204, surface layer protein) was synthesized and inserted into pUC55 (pUC55-*NlpC* was constructed by BGI company, Shanghai, China). Then the obtained plasmid pUC55-*NlpC* was used as a template to amplify the *NlpC* gene using FastPfu DNA polymerase (TransGen Biotech, Beijing, China) and *NlpC*-F-SOE and *NlpC*-R primers (PCR1, Table 3). The DNA sequence coding for His tag (in italics) and partial multiple cloning sites (MCS, in bold) were included in the *NlpC*-R primer. The *NlpC* gene, His-tag gene, and partial MCS were arranged in a row within the PCR1 product, and the PCR1 product was named as *NlpC*-His-MCS (*NHM*) gene. Meanwhile, the *P*_*slyA*_ gene was amplified using pELX1 and FastPfu DNA polymerase with *P*_*slyA*_-F and *P*_*slyA*_-R-SOE primers (PCR2, Table 3). The splicing overlapping extension (SOE) PCR (amplification composition shown in Additional file 1: Table S1) [28] was used to fuse the *P*_*slyA*_ gene and *NHM* gene using *P*_*slyA*_-F and *NlpC*-R primers (Table 3). The SOE-PCR product and pELX1 vector were digested with EcoRI and BglП at 37 °C for 4 h, and then purified by Cycle-Pure Kit (Omega Bio-Tek, USA) and Gel Purification Kit (TIANGEN, China), respectively. The digested products were linked using T4 DNA ligase (Thermo Scientific, USA) for constructing the expression plasmid pELCW.

**Table 3:** Specific PCR used in this study

Genes	Primers	Amplification parameters
*NHM*(PCR1)	*NlpC*-F-SOE: CAAGGAGGAAAAGACCACATGGTAGATGCAAAGAAAGTATTG	95 °C for 5 min, 95 °C for 30 s, 57 °C for 30 s, and 72 °C for 60 s
	*NlpC*-R: GGA**AGATCTCCATGGCTCGAG***ATGATGATGATGATGGTGTAG*TGAAGGACGAACAGC	
*P*_*slpA*_(PCR2)	*P*_*slpA*_-F: CCGGAATTCAAGCGGTAGGTG	95 °C for 5 min, 95 °C for 30 s, 57 °C for 30 s, and 72 °C for 40 s
	*P*_*slpA*_-R-SOE: CAATACTTTCTTTGCATCTACCATGTGGTCTTTTCCTCCTTG	
*P*_*slpA*_-*NHM*(SOE-PCR)	*P*_*slpA*_-F: CCGGAATTCAAGCGGTAGGTG	95 °C for 5 min, 95 °C for 30 s, 56 °C for 30 s, and 72 °C for 100 s
	*NlpC*-R: GGA**AGATCTCCATGGCTCGAG***ATGATGATGATGATGGTGTAG*TGAAGGACGAACAGC	
*aiiK*(PCR3)	*aiiK*F: GGAAGATCTATGTGTCAAAATAAAAAGTTGTAC	95 °C for 5 min, 95 °C for 30 s, 54 °C for 30 s, and 72 °C for 60 s
	*aiiK*R: CGGGGTACCTTATTCGTAATACCCTTCCGTTGA	
Sequencing	EcoRI-*P*_*SlpA*_-F: CCGGGAATTCAAGCGGTAGGTGAAATATTAC	95 °C for 5 min, 95 °C for 30 s, 55.5 °C for 30 s, and 72 °C for 120 s
	BamHI-T-R: GGCCGGATCCAGCTTGCGTTTGATTTTC	

His tag was marked in italics, and multiple cloning sites (MCS) were marked in bold

The genomic DNA of *K. huakuii* LAM0618^T^ was utilized to amplify the *aiiK* gene using the *aiiK*F and *aiiK*R primers (PCR3, Table 3). The *aiiK* gene and pELCW vector were digested with BglП and KpnI at 37 °C for 4 h, and purified as mentioned earlier. Then ligation was performed by T4 DNA ligase to form pELCW-*aiiK*. Both pELCW and pELCW-*aiiK* were transformed into *E. coli* DH5α for storage, and then sequenced by company BGI (Shanghai, China) with the EcoRI-*P*_*SlyA*_-F and BamHI-T-R primers (Table 3).

### Construction of the recombinant strain of pELCW-*aiiK*/*L. casei* MCJΔ1

Competent cells of *L. casei* MCJΔ1 were prepared based on our previous study [27]. Electrotransformation was performed as follows: 200 ng of pELCW-*aiiK* or pELCW was mixed with the 80-μL competent cells. The mixture was transferred into a 2-mm electroporation cuvette (Bio-Rad, USA), and then incubated on ice for 10 min. Subsequently, the electroporation was carried out at 1500 V and 5 ms with an Eppendorf Multiporator (Eppendorf, Hamburg, Germany). After electroporation, the mixture was transferred to 920-μL pre-warmed MRSS (MRS with 0.3 M sucrose) broth, and then incubated at 37 °C for 3 h for recovery. At last, the mixture was plated on MRS agar with erythromycin and incubated at 37 °C for 48 h to screen for recombinant strains.

### Detection of AHL lactonase activity of *Lc*AiiK

*Lactobacillus casei* MCJΔ1 harboring pELCW-*aiiK* was inoculated into 100 mL of fresh MRS medium (containing erythromycin) at the ratio of 1%. After incubation at 37 °C for 20 h, the cells were harvested by centrifugation and washed twice with 10 mM phosphate buffer saline (PBS, pH 7.4). These washed cells were re-suspended in 10 mM PBS, and the suspension was subjected to detect the AHL lactonase activity. The reaction mixture (500 μL) containing *Lc*AiiK cells (OD_600_ = 5.0) and 50 μM *N*-Hexanoyl-l-homoserine lactone (C_6_-HSL) in 10 mM PBS (pH 7.4) was incubated at 37 °C for 3 h. Then, sodium dodecyl sulfate (SDS) was added into the mixture to terminate the reaction. The unreacted C_6_-HSL was extracted based on our previous study [18]. For the negative control, *Lc*AiiK cells were replaced by pELCW/*L. casei* MCJΔ1 (*Lc*CW) cells with the other conditions being the same. For the positive control, 4 μg/μL of AiiK purified from *E. coli* BL21 (DE3) was used to replace *Lc*AiiK cells. At last, the extracted C_6_-HSL was detected by using the violacein generation bioassay and quantified using the high performance liquid chromatography (HPLC).

In the violacein generation bioassay, 1 mL overnight culture of *C. violaceum* CV026 was mixed well with 24 mL molten LB agar (1.6%), and the mixture was poured onto the plates. After the agar solidification, a sterile filter paper disk with a diameter of 5.5 mm was placed on every plate. The extracted C_6_-HSL samples were dropped onto the filter paper discs, and the plates were incubated at 30 °C for 16 h to generate violacein.

In the HPLC analysis, the Agilent Eclipse Plus C18 (4.6 × 250 mm, 5 μm) column and Agilent Technologies 1200 series were employed. The extracted C_6_-HSL was separated at 22 °C with a constant flow rate of 0.7 mL/min in isocratic elution (acetonitrile/water = 31/69, v/v) and then detected at 210 nm.

### Localization assays of AiiK in *Lc*AiiK

The surface layer proteins are localized on the outer layer of the peptidoglycan, lysozyme can degrade the peptidoglycan of gram-positive bacterial cell wall, and release the surface layer proteins. To verify that the AiiK was expressed on the surface layer of *Lc*AiiK cells, the localization of AiiK was carried out. *Lc*AiiK cells (OD_600_ = 0.5) were treated with lysozyme (Amresco, China) (20 mg/mL) in 10 mM PBS (pH 7.4) at 37 °C for 1 h, 2 h, and 3 h. After incubation, the mixture was centrifuged at 12,000 rpm for 2 min to collect the supernatant for detecting AHL lactonase activity. The substrate C_6_-HSL (50 μM) was added into and incubated at 37 °C for 12 h. The unreacted C_6_-HSL was quantified using the method described above. For the positive control, the supernatant was replaced with *Lc*AiiK cells (OD_600_ = 0.5). For the negative control, the supernatant was replaced with the same volume of lysozyme solution (lysozyme was dissolved in 10 mM PBS, pH 7.4).

### Characteristics of *Lc*AiiK

The characteristics of *Lc*AiiK were determined by detecting its AHL lactonase activity. The reaction mixture (500 μL) contained *Lc*AiiK cells (OD_600_ = 0.5) and C_6_-HSL (50 μM) in PBS (10 mM, pH 7.4). In C_6_-HSL degradation assay, the reaction mixture was incubated at 37 °C for 0, 1, 2, 4, 6, 8, 10, 12, 16, 20, and 24 h, respectively, and the residual C_6_-HSL was quantified by HPLC. The optimal cell density (OD_600_) of *Lc*AiiK to degrade C_6_-HSL was determined using different optical densities (OD_600_ = 0.1, 0.5, 1.0, 2.0, and 3.0), and the reaction mixture was incubated at 37 °C for 12 h. The optimal reaction temperature was determined by incubating the reaction mixture at 25 °C, 30 °C, 35 °C, 37 °C, 40 °C, 45 °C, and 50 °C. The effects of divalent metal ions on *Lc*AiiK in vivo were examined by cultivating *Lc*AiiK in DMIF-MRS broth with addition one kind of divalent metal ions (1 mM Zn^2+^, 1 mM Mg^2+^, 1 mM Mn^2+^, 1 mM Co^2+^, and 1 mM Ni^2+^), the cultures were incubated at 37 °C for 20 h. After incubation, the cells were harvested and subjected to detect AHL lactonase activity. The effects of divalent metal ions on *Lc*AiiK in vitro were examined by culturing *Lc*AiiK in DMIF-MRS broth at 37 °C for 20 h. After incubation, the harvested cells and one kind of divalent metal ions (1 mM Zn^2+^, 1 mM Mg^2+^, 1 mM Mn^2+^, 1 mM Co^2+^, and 1 mM Ni^2+^) were added to detect the AHL lactonase activity.

### Determination of plasmid stability

To calculate the plasmid stability of pELCW-*aiiK* in strain *L. casei* MCJΔ1, the *Lc*AiiK was inoculated into MRS at ratio of 1% without erythromycin for continuous passage culture of 15 generations, and every generation was propagated for 12 h. At every generation, colony-forming units (cfu) were determined by MRS agar plates and selective MRS agar plates (50 μg/mL erythromycin). The plasmid stability of pELCW-*aiiK* was calculated as the ratio of cfu number on selective MRS agar versus that on MRS agar [27]. Therefore, the plasmid stability per generation was calculated by equation:

$$L=\sqrt[n]{\bar{x}/\bar{y}}\;{\times}\;100\%$$

Herein, “*L*” is plasmid stability, “*n*” is continuous generations cultured, “${\bar{x}}$” is the average cfu numbers on selective MRS agar, and “${\bar{\text{y}}}$” is the average cfu numbers on MRS agar.

### Effect of *Lc*AiiK on swimming motility in *A. hydrophila*

The effect of *Lc*AiiK on swimming motility in *A. hydrophila* was determined based on the method described by Jahid et al. with minor modifications [6]. Fifty microliter of co-culture mixture containing *Lc*AiiK at various concentrations (OD_600_ = 0.1, 0.2, and 0.4) and *A. hydrophila* AH-1 or AH-4 (OD_600_ = 0.1) in 10 mM PBS (pH 7.4) was inoculated onto the center of NA (NB with 0.3% agar) plates, and then incubated at 25 °C for 24 h. After incubation, the diameter of strain lawn was measured. The *Lc*CW cells was used as negative control, and 10 mM PBS was used as control check (CK).

### Effect of *Lc*AiiK on haemolytic activity in *A. hydrophila*

Blood agar plates were utilized to evaluate the effect of *Lc*AiiK on haemolytic activity in *A. hydrophila* [29]. Eighty microliter of co-culture mixture, comprising *Lc*AiiK (OD_600_ = 0.5) and *A. hydrophila* AH-1 or AH-4 (OD_600_ = 0.1) in 10 mM PBS (pH 7.4), was inoculated into the hole on blood agar plate and incubated at 30 °C for 24 h. The zone of complete haemolysis was measured to assess haemolytic activity of *A. hydrophila*. The negative control and CK were prepared as mentioned earlier.

### Effect of *Lc*AiiK on extracellular proteolytic activity in *A. hydrophila*

The effect of *Lc*AiiK on extracellular proteolytic activity in *A. hydrophila* was evaluated by conducting an extracellular proteolytic assay according to Bhakti et al. with modifications [30]. *A. hydrophila* AH-1 or AH-4 was inoculated into fresh NB with different concentrations of *Lc*AiiK (OD_600_ = 0.1, 0.2, and 0.4), then the mixture was incubated at 30 °C and 180 rpm for 20 h. After incubation, the NB culture supernatant was used as the crude enzyme extract for detecting the extracellular proteolytic activity. The reaction mixture contained 250 μL of supernatant and 250 μL of 2% azocasein. After reacting at 30 °C for 3 h, 1.2 mL 10% trichloroacetic acid was added and centrifuged at 6000 g for 10 min. Then 1.2 mL of supernatant was mixed with 1.0 mL of 1 M NaOH, and the optical density was assessed at 440 nm. The negative control and CK were prepared as mentioned earlier.

### Effect of *Lc*AiiK on biofilm formation by *A. hydrophila*

The effect of *Lc*AiiK on the biofilm formation by *A. hydrophila* was examined based on the method described by Dong et al. with some modifications [18]. About 200 μL co-culture mixture, containing *Lc*AiiK with various concentrations (OD_600_ = 0.1, 0.2, and 0.4) and *A. hydrophila* AH-1 or AH-4 (OD_600_ = 0.1), was dispensed into a 96-well microtiter plate and statically incubated at 30 °C for 12 h. After incubation, planktonic cells from the plate were transferred out gently for dilution plate count of *A. hydrophila* AH-1 and AH-4. The biofilm cells were washed very gently with 10 mM PBS for three times, and then stained with 20 μL of 0.2% crystal violet at 25 °C for 15 min. The stained biofilm cells were washed very gently with distilled water for three times. Ethanol (100 μL, 95%) was added to extract crystal violet, and the absorbance at 590 nm was measured. The negative control and CK were prepared as mentioned earlier.

### Statistical analysis

All data were processed using Excel (version 2019) as mean ± standard deviation (sd), and differences with P < 0.05 and P < 0.01 were deemed significant.

## Results

### Construction of recombinant strain *Lc*AiiK

**Fig. 1:**
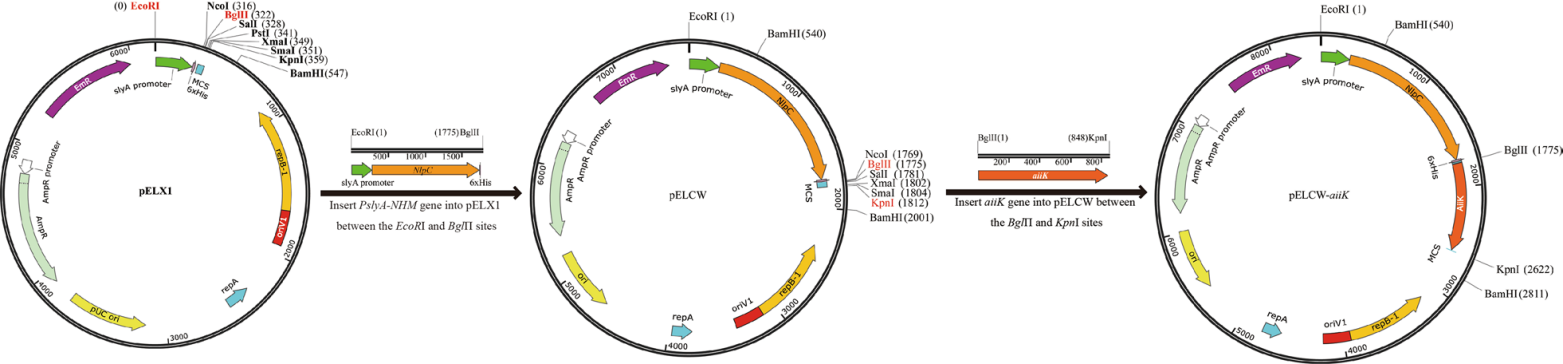
Flow chart of construction of the cell wall-associated expression vectors pELCW and pELCW-*aiiK*

To construct the cell wall-associated expression vector pELCW, we inserted gene *NlpC* at the end of promoter *P*_*slyA*_ and in the front of His-tag gene on plasmid pELX1. The *NlpC* gene was used as a guide peptide sequence to help target protein anchor at the surface layer. The SOE-PCR product (*P*_*slyA*_-*NHM* gene) (Additional file 1: Fig. S1) was inserted into pELX1 between the EcoRI and BglП sites (Fig. 1). We named the new plasmid pELCW, which was constructed from pELX1 with cell wall-associated expression function. Furthermore, the *aiiK* gene was inserted into pELCW between the BglП and KpnI sites (Fig. 1), generating the plasmid pELCW-*aiiK* (Fig. 1).

Screening of the recombinant strain by colony PCR and plasmid sequencing indicated that the pELCW-*aiiK*/*L. casei* MCJΔ1 and pELCW/*L. casei* MCJΔ1 were constructed correctly. The recombinant strain pELCW-*aiiK*/*L. casei* MCJΔ1 was designated as *Lc*AiiK (AiiK expressed by *L*. *casei* MCJΔ1) whereas the recombinant strain pELCW/*L. casei* MCJΔ1 was designated as *Lc*CW.

### Detection of AHL lactonase activity of *Lc*AiiK and localization assays of AiiK in *Lc*AiiK

**Fig. 2:**
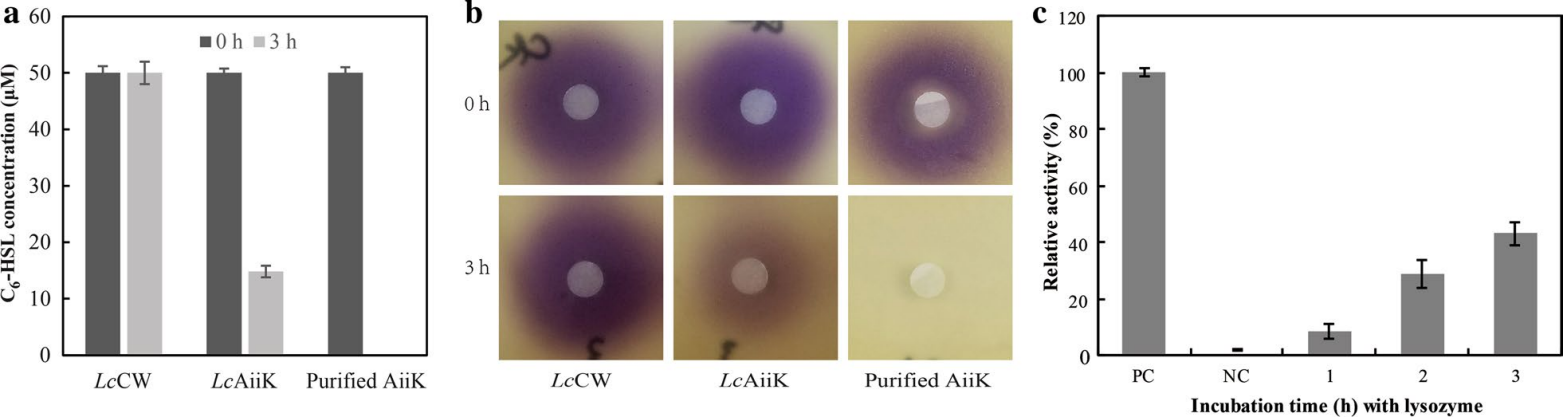
**a** AHL lactonase activity of *Lc*AiiK detected by HPLC (*Lc*CW cells were used as negative control, purified AiiK was used as positive control). **b** AHL lactonase activity of *Lc*AiiK detected by violacein generation of *C. violaceum* CV026 (*Lc*CW cells were used as negative control, purified AiiK was used as positive control). **c** Localization assays of AiiK in *Lc*AiiK, PC represents positive control and NC represents negative control. Data are shown as mean ± SD, n = 3

The results showed that *Lc*AiiK (OD_600_ = 5.0) could degrade 35.18 μM C_6_-HSL at 37 °C within 3 h and *Lc*CW didn’t exhibit any AHL lactonase activity to C_6_-HSL (Fig. 2a). The purified AiiK (4 μg/mL) from *E .coli* BL21 (DE3) degraded 50 μM C_6_-HSL at 37 °C within 3 h (Fig. 2a). Meanwhile, the same results were detected by violacein generation bioassay of *C. violaceum* CV026 (Fig. 2b). Here, *Lc*AiiK cells (without any treatment) were used to directly degrade C_6_-HSL, and *Lc*AiiK cells did degrade C_6_-HSL (Fig. 2a). From this, we can speculate that AiiK was expressed and located at the outermost layer of *Lc*AiiK cells. Therefore, *Lc*AiiK exhibited significant AHL lactonase activity, and the protein AiiK was expressed at the outermost layer of *Lc*AiiK cells (Fig. 2a and b).

Although the protein AiiK was expressed at the outermost layer of *Lc*AiiK cells, it remained unclear whether AiiK was expressed at surface layer of *Lc*AiiK. The localization assays of AiiK revealed that AHL lactonase activity of supernatant increased with incubation time compared with negative control (Fig. 2c). The lysozyme can degrade peptidoglycan of cell wall and release surface layer proteins. Thus, AHL lactonase activity of supernatant increased significantly within the treatment of lysozyme, which indicated that AiiK was expressed at the surface layer of *Lc*AiiK.

### Characteristics of *Lc*AiiK

**Fig. 3:**
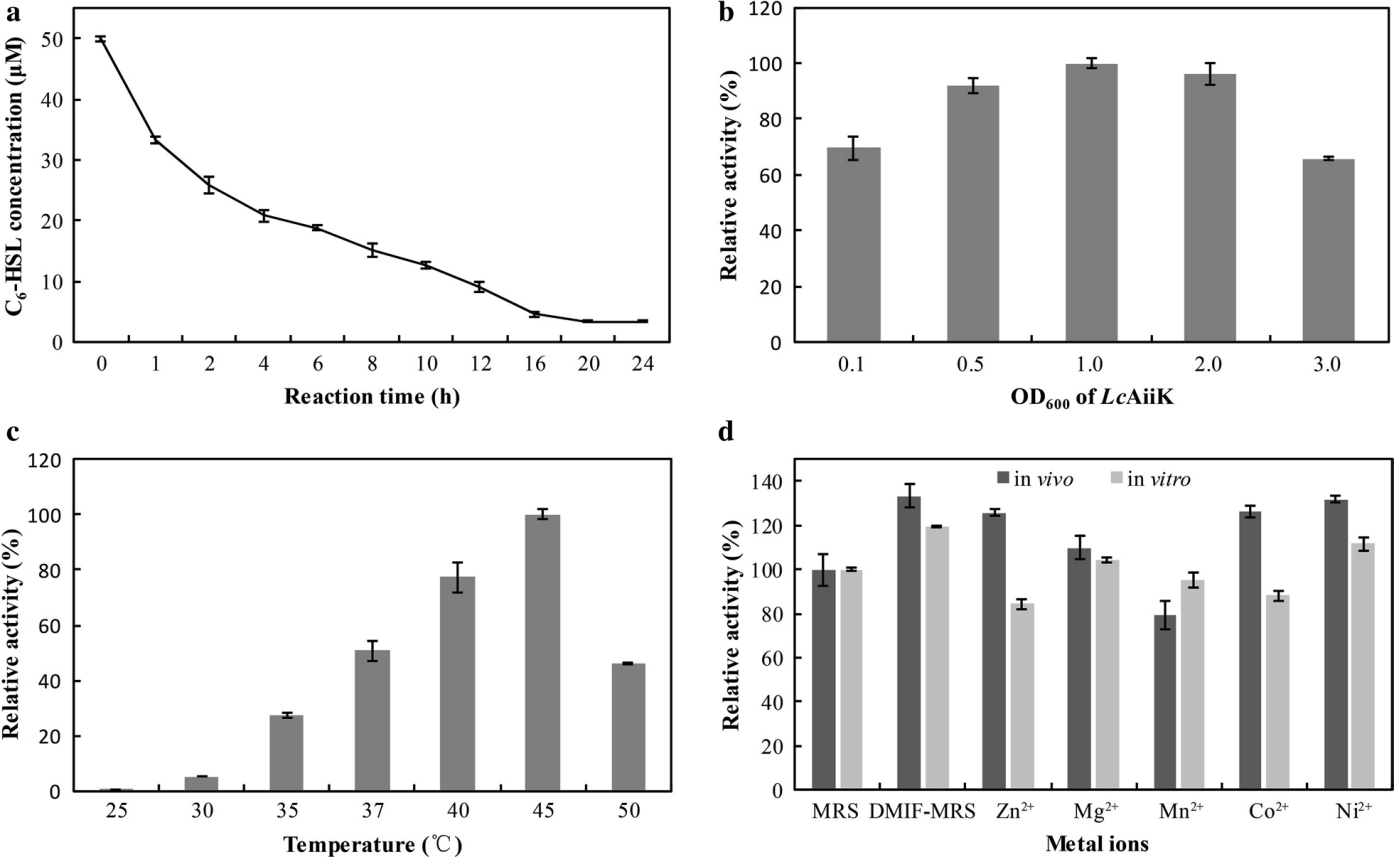
Characteristics of *Lc*AiiK. **a** C_6_-HSL degradation curve of *Lc*AiiK within 24 h. **b** Optimal OD_600_ of *Lc*AiiK for degrading C_6_-HSL. **c** Optimal reaction temperature of *Lc*AiiK. **d** Effect of divalent metal ions on AHL lactonase activity of *Lc*AiiK in vivo and vitro [reaction was performed with *Lc*AiiK cells (OD_600_ = 0.5) at 37 °C for 12 h]. Data are shown as mean ± SD, n = 3

Many previous studies have reported that C_6_-HSL is a vital signal molecule mediating QS processes such as motility, haemolytic activity, extracellular proteolytic activity, and biofilm formation in *A. hydrophila* [6, 26, 31–33]. Thus, C_6_-HSL was used as substrate to detect AHL lactonase activity of *Lc*AiiK in our study. *Lc*AiiK (OD_600_ = 0.5) degraded 24.13 μM of C_6_-HSL at 2 h, 40.99 μM of C_6_-HSL at 12 h, and 46.63 μM of C_6_-HSL at 24 h (Fig. 3a). The optimal OD_600_ of *Lc*AiiK to degrade C_6_-HSL was determined at value of 1.0 (Fig. 3b). The optimal reaction temperature of *Lc*AiiK to degrade C_6_-HSL was 45 °C (Fig. 3c). The in vivo experiments showed that Zn^2+^, Mg^2+^, Co^2+^, and Ni^2+^ increased AHL lactonase activity of *Lc*AiiK, however, Mn^2+^ slightly decreased its activity (Fig. 3d). Moreover, the in vitro experiments revealed that Mg^2+^ and Ni^2+^ increased AHL lactonase activity of *Lc*AiiK, however, Zn^2+^, Mn^2+^, and Co^2+^ decreased its activity (Fig. 3d). Interestingly, *Lc*AiiK cultured in DMIF-MRS was found to exhibit higher AHL lactonase activity than that cultured in MRS from both in vivo and in vitro experiments (Fig. 3d). These characteristics provided the foundation and guiding significance for the practical use of *Lc*AiiK.

### Determination of plasmid stability

**Fig. 4:**
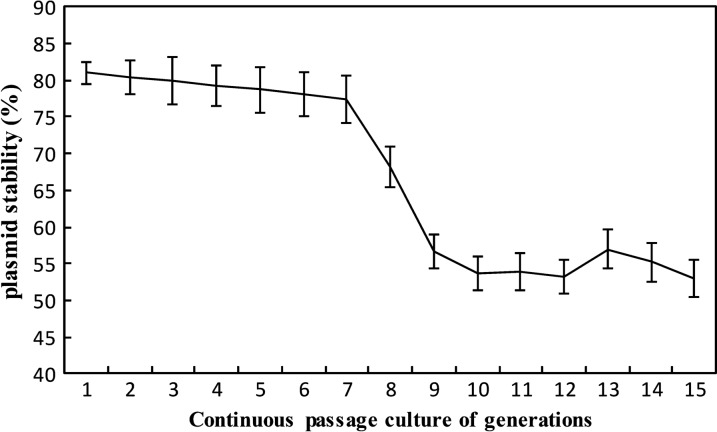
pELCW-*aiiK* plasmid stability of *Lc*AiiK during continuous passage culture in MRS for 15 generations under the nonselective condition. Data are shown as mean ± SD, n = 3

The plasmid stability of pELCW-*aiiK* was measured by counting the colonies after continuous passage culture in MRS for 15 generations under the nonselective condition. The results revealed that plasmid stability of pELCW-*aiiK* decreased slightly from 81.00% to 77.38% during the first 7 generations, then tobogganed to 56.69% at generation 9, and finally remained stable from generation 10 to 15 with the value of 53.04% (Fig. 4). In short, over 50% of *Lc*AiiK cells maintained plasmid pELCW-*aiiK* after 15 generations without erythrocin. From this result, we speculated that the *Lc*AiiK might maintain the QQ ability for a period which was sufficient for *Lc*AiiK cells to maintain the pELCW-*aiiK* plasmid while proliferating.

### *Lc*AiiK’s application in QQ on *A. hydrophila*

**Fig. 5:**
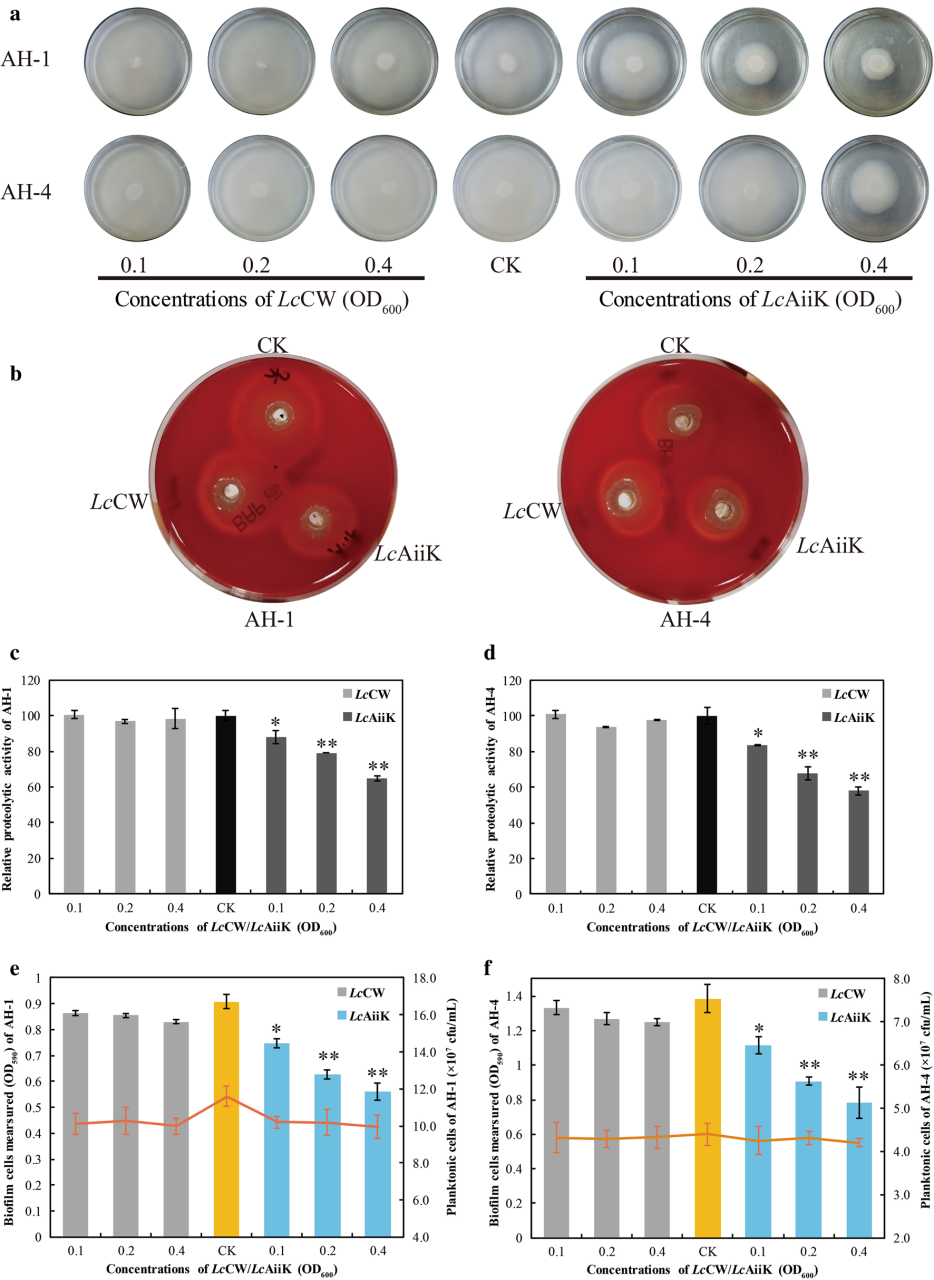
Effect of *Lc*AiiK on the swimming motility, virulence factor production, and biofilm formation in *A. hydrophila* AH-1 and AH-4. **a** The swimming motility of *A. hydrophila* AH-1 and AH-4. **b** The haemolytic activity of *A. hydrophila* AH-1 and AH-4. **c** The extracellular proteolytic activity of *A. hydrophila* AH-1. **d** The extracellular proteolytic activity of *A. hydrophila* AH-4. **e** Biofilm formation (column chart) was detected by crystal violet staining, and planktonic cells (red line chart) were detected by plate counting of *A. hydrophila* AH-1. **f** Biofilm formation (column chart) was detected by crystal violet staining, and planktonic cells (red line chart) were detected by plate counting of *A. hydrophila* AH-4. Data are shown as mean ± SD, n = 3. A *t* test was performed for testing differences between groups, and the ** and * indicate P < 0.01 and P < 0.05, respectively

*Lc*AiiK was found to quench the AHL-mediated QS processes of *A. hydrophila* AH-1 and AH-4 in this study (Fig. 5). *Lc*AiiK significantly hampered the swimming motility of *A. hydrophila* AH-1 and AH-4, compared to CK and negative control (Fig. 5a). The inhibition ratio of swimming motility was dependent on the dose of *Lc*AiiK (Fig. 5a). *Lc*AiiK showed slight inhibition effect on haemolytic activity of *A. hydrophila* AH-1 and AH-4, compared to CK and negative control (Fig. 5b). *Lc*AiiK (OD_600_ = 0.4) reduced extracellular proteolytic activity of *A. hydrophila* AH-1 and AH-4 by 35.29% (Fig. 5c) and 42.01% (Fig. 5d) after co-culture, respectively, and *Lc*CW (negative control) did not exhibit inhibition effect on that of *A. hydrophila* AH-1 (Fig. 5c) and AH-4 (Fig. 5d). Moreover, the increased application of *Lc*AiiK resulted in the obvious decrease in biofilm formation of *A. hydrophila* AH-1 (Fig. 5e) and AH-4 (Fig. 5f), compared to CK and negative control. Furthermore, plate counting results indicated that planktonic cells of *A. hydrophila* AH-1 and AH-4 remained stable at the value of 10.1 × 10^7^ cfu/mL (Fig. 5e) and 4.3 × 10^7^ cfu/mL (Fig. 5f) after co-culture, respectively. And this reflected that *Lc*AiiK inhibited biofilm formation of *A. hydrophila* AH-1 and AH-4, but not kill them when co-culture. Therefore, these results demonstrated that *Lc*AiiK obviously attenuated the swimming motility, haemolytic activity, extracellular proteolytic activity, and biofilm formation of *A. hydrophila* AH-1 and AH-4.

## Discussion

QS is a population-dependent behavior in bacteria for communicating with each other, which orchestrates expression of multiple genes triggered by signal molecules when external environment changes [8, 10, 34–38]. AHLs as important signal molecules mediating many QS processes were identified in multiple gram-negative bacterial species, and most bacteria were common pathogens existing in various environments [26]. Many studies have revealed that AHL-mediated QS was closely related to the pathogenicity, virulence factor production, and biofilm formation in gram-negative pathogens [2, 3]. The acute hemorrhagic septicemia in fish and diarrhea even death in human caused by microbial infections were closely related to the AHL-mediated QS in various gram-negative pathogens, especially *A. hydrophila*, *A. salmonicida*, and *P. aeruginosa* [4, 5, 39–42]. However, the antibiotic therapy for these gram-negative pathogens will accelerate the emergence of drug-resistance. Thus, it is urgent to develop a promising strategy to inhibit or quench these processes of AHL-mediated QS [43–45]. Interfering or quenching QS, known as QQ, is becoming a prospective tactic for reducing the pathogenicity triggered by AHL-mediated QS [9, 17–20, 46, 47]. QQ enzymes are of great importance in inhibiting or attenuating pathogenicity. Therefore, the QQ enzymes can be widely applied as bio-control agents.

In this study, QQ enzyme AiiK was expressed at the surface layer of strain *L. casei* MCJΔ1. This is the first report that QQ enzyme was expressed in lactic acid bacteria *Lactobacilli* genus implemented by cell wall-associated constitutive expression vector pELCW-*aiiK*. Meanwhile, the cell wall-associated constitutive expression vector pELCW provides a genetic tool for DNA clone and a new perspective for heterologous gene expression at the surface layer of strain *L. casei* MCJΔ1. Many studies have reported that QQ enzymes were heterologously expressed by *E. coli*, but few works were done by utilizing other expression systems. So far, Chen et al. used the vector pPIC9 to express recombinant AiiA_B546_ in *Pichia pastoris* [21], and Zhang et al. applied *B. brevis* expression system to express MomL [26]. These two expression systems produced secreted proteins and the application of the proteins required prior purification steps. Herein, we utilized vector pELCW-*aiiK* to express the AiiK which anchored at the surface layer of *Lc*AiiK, making *Lc*AiiK cells can be applied directly without any processing steps.

Considering the characteristics of *Lc*AiiK, our results revealed that *Lc*AiiK maintained the same optimal reaction temperature with purified AiiK at 45 °C [18]. The effect of divalent metal ions on *Lc*AiiK was slighter than that on purified AiiK [18]. Based on this finding, it could be speculated that *Lc*AiiK is less affected by external environment (such as divalent metal ions) than purified AiiK. AHL lactonase activity of *Lc*AiiK increased from 100.00% (day 1) to 192.10% (day 3), then dropped slowly to 155.87% (day 6) (Additional file 1: Fig. S2). However, the live *Lc*AiiK cells were decreased quickly from 7.4 × 10^7^ cfu/mL (day 1) to 7.8 × 10^6^ cfu/mL (day 6) when *Lc*AiiK was stored in 10 mM PBS at 4 °C (Additional file 1: Fig. S2). Based on this result, we speculated that the cell wall lysis released the AiiK from surface layer and increased AHL lactonase activity (Additional file 1: Fig. S2). *Lc*AiiK could maintain 155.87% AHL lactonase activity after 6-day storage at 4 °C (Additional file 1: Fig. S2), while purified AiiK could only retain 20% AHL lactonase activity after 12-h storage at 37 °C [18]. This finding implied that *Lc*AiiK endows higher stability compared to that of purified AiiK, which solves the drawback of instability of purified AiiK. The anchored AiiK at the surface layer of *Lc*AiiK maintained good stability to sustainably degrade AHLs (Additional file 1: Fig. S2), which can be more useful in factual environment. Moreover, *Lc*AiiK could retain over 50% plasmid stability of pELCW-*aiiK* within 15 generations, reflecting that plasmid pELCW-*aiiK* can exist in cells for a long time. Our previous study reported that AiiK could degrade multiple AHLs including C_6_-HSL, 3-Oxo-C_6_-HSL, C_10_-HSL, and C_14_-HSL [18]. Therefore, these characteristics of *Lc*AiiK lay a solid foundation for its application in the field of QQ.

**Fig. 6:**
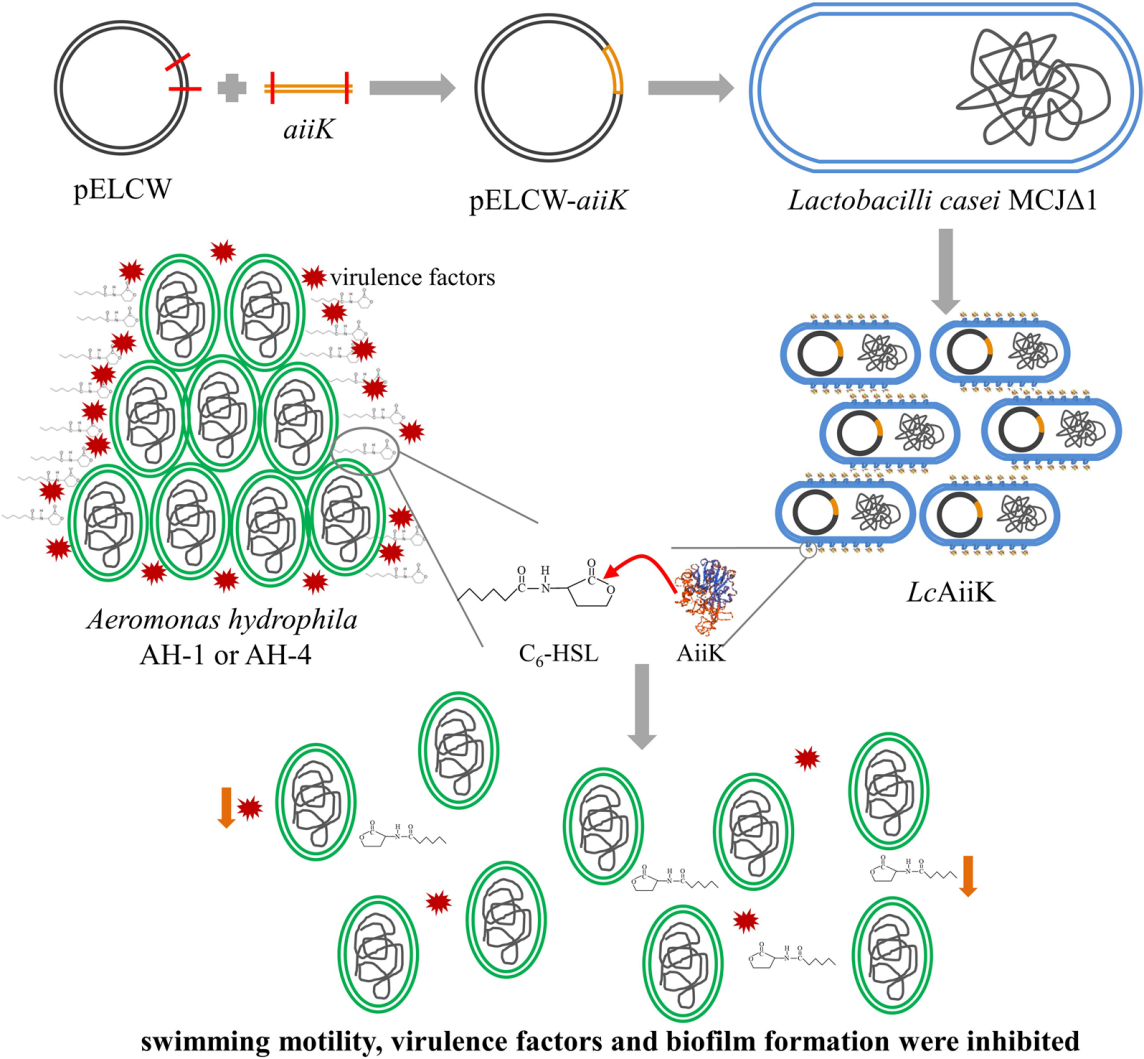
Schematic diagram of QQ mechanism of *Lc*AiiK. The expression of AiiK at the surface layer of *Lc*AiiK maintained AHL lactonase activity. *Lc*AiiK can degrade C_6_-HSL produced by *A. hydrophila* and interrupt or inhibit the production of virulence factors of *A. hydrophila*

Herein, we verified the QQ ability of *Lc*AiiK against *A. hydrophila* by co-culture. The reason why *A. hydrophila* was selected as the target strain was that this bacterium is an emerging gram-negative opportunistic pathogen that can cause various serious symptoms in fish, crabs, shrimps, and even humans [4, 6, 7]. Many studies have reported that the pathogenicity and human infections depend on the AHL-mediated QS in *A. hydrophila* [6, 13]. The main AHL signal molecules are C_6_-HSL and *N*-butanoyl-L-homoserine lactone (C_4_-HSL) in *A. hydrophila* [6, 13, 31–33]. Besides, the biofilm formation of *A. hydrophila* was highly associated with multiple antibiotics resistance, making the diseases or infections difficult to be cured [14]. In this study, the AiiK was expressed at the surface layer of *Lc*AiiK with AHL lactonase activity (Fig. 2). *Lc*AiiK exhibited an obvious QQ ability against *A. hydrophila* AH-1 and AH-4 by degrading their signal molecule C_6_-HSL (Figs. 5 and Fig. 6). Meanwhile, *Lc*AiiK did not kill the planktonic cells of *A. hydrophila* AH-1 and AH-4 at co-culture condition, implying that *Lc*AiiK did not accelerate the emergence of drug-resistance (Fig. 5e and f). Therefore, this might be a promising anti-pathogenic strategy to control pathogenic bacteria and to prevent antibiotic resistance. It was reported that recombinant strain *Bb*MomL significantly reduced the secretion of pathogenic factors and the pathogenicity of *P. carotovorum* subsp. carotovorum and *P. aeruginosa* PAO1 [26]. Chen et al. expressed QQ enzyme AiiA_B546_ by pPIC9/*P. pastoris* expression system, and AiiA_B546_ decreased the mortality rate and delayed the mortality time of fish by co-injecting *A. hydrophila* and AiiA_B546_ into common carp [21]. In our study, the AiiK was expressed at the surface layer of *Lc*AiiK. Therefore, *Lc*AiiK cells harboring the AiiK protein on their cell walls were co-cultured directly with *A. hydrophila* AH-1 and AH-4 to quench their QS processes. This strategy is easy to apply as it only involves cultivation of *Lc*AiiK cells without purification steps. Zhou et al. reported that *Bacillus* sp. QSI-1 significantly decreased haemolytic and protease activity of *A. hydrophila* YJ-1 [48], which was consistent with our present results.

Interestingly, *Lc*AiiK was found to exhibit higher AHL lactonase activity when it was cultured in DMIF-MRS than in MRS (Fig. 3d). Correspondingly, we speculated that the AHL lactonase activity of *Lc*AiiK could be improved by optimizing the culture formula (such as divalent metal ions), and this speculation deserves further study. As for safety issue, *L. casei* is one kind of lactic acid bacteria which is commonly considered as an environment friendly probiotic. Furthermore, many studies have demonstrated that the *L. casei* is an important probiotic [49–52]. *L. casei* inhibited the growth of *Streptococcus mutans* in caries prevention [49], decreased the relative abundance of intestinal *Escherichia*-*Shigella* in suckling rabbit [50], and attenuated the biofilm development of *Candida albicans* [51]. The health-promoting feature of *L. casei* was documented by Hill et al., reporting the potentials of *L. casei* in the treatment or prevention of a variety of diseases [52]. Therefore, the probiotic capabilities of *L. casei* made *Lc*AiiK safe to be applied. These probiotic capabilities also expanded the application range of *Lc*AiiK, which suggested that *Lc*AiiK could not only be used as probiotic, but also be exploited as an anti-pathogenic drug or a bio-control agent against the AHL-mediated QS pathogenic bacteria.

## Conclusions

AHL lactonase AiiK is firstly expressed at the surface layer of *L. casei* MCJΔ1 via a cell wall-associated constitutive expression vector pELCW-*aiiK*. *Lc*AiiK exhibited considerable AHL lactonase activity and displayed obvious QQ ability against *A. hydrophila* AH-1 and AH-4 by attenuating their swimming motility, virulence factor production, and biofilm formation instead of killing them. Therefore, the *Lc*AiiK can be developed as an anti-pathogenic agent to control AHL-mediated QS pathogenic bacteria and prevent the emergence of antibiotic resistance.

## Supplementary information

**Additional file 1.** **Text 1.** 16S rDNA sequence of* A. hydrophila* AH-1. **Text 2.** 16S rDNA sequence of* A. hydrophila* AH-4. **Table S1.** Amplification composition and condition of SOE-PCR.** Fig. S1.** Agarose gel electrophoresis of SOE-PCR product* P*_*slyA*_-*NHM* gene. **Fig. S2.** AHL lactonase activity and live cells of *Lc*AiiK after storage at 4 °C.

## Data Availability

All data generated or analyzed during this study are included in this published article and its Additional file.
