# Supplementary material for: Heterologous expression of AHL lactonase AiiK by Lactobacillus casei MCJΔ1 with great quorum quenching ability against Aeromonas hydrophila AH-1 and AH-4
Source: Microb Cell Fact. 2020 Oct 7;19:191. doi: 10.1186/s12934-020-01448-4 (PMC7542731; doi:10.1186/s12934-020-01448-4)
Supplement: Supplementary file 1 — Additional file 1. Text 1. 16S rDNA sequence of A. hydrophila AH-1. Text 2. 16S rDNA sequence of A. hydrophila AH-4. Table S1. Amplification composition and condition of SOE-PCR. Fig. S1. Agarose gel electrophoresis of SOE-PCR product PslyA-NHM gene. Fig. S2. AHL lactonase activity and live cells of LcAiiK after storage at 4 °C. [file 12934_2020_1448_MOESM1_ESM.docx]

**Supplementary material for**

**Heterologous expression of AHL lactonase AiiK by *Lactobacillus casei* MCJΔ1 with great quorum quenching ability against *Aeromonas hydrophila* AH-1 and AH-4**

Weiwei Dong^1^, Yuyuan Cai^1^, Zhilong Xu^1^, Biao Fu^1^, Qitong Chen^1^, Yuxin Cui^1^, Zhiyong Ruan^2^, Yunxiang Liang^1^, Nan Peng^1^, Shumiao Zhao^1^*

^1^ State Key Laboratory of Agricultural Microbiology and College of Life Science and Technology, Huazhong Agricultural University, Wuhan 430070, China.

^2^ Institute of Agricultural Resources and Regional Planning, CAAS, Beijing 100081, China.

* Corresponding author: Shumiao Zhao

Tel: +86 27 87281040; Fax: +86 27 87280670

E-mail: [shumiaozhao@mail.hzau.edu.cn](mailto:shumiaozhao@mail.hzau.edu.cn)

>16S rDNA sequence of *A. hydrophila* AH-1

taccatgcagtcgagcggcagcgggaaagtagcttgctacttttgccggcgagcggcggacgggtgagtaatgcctgggaaattgcccagtcgagggggataacagttggaaacgactgctaataccgcatacgccctacgggggaaagcaggggaccttcgggccttgcgcgattggatatgcccaggtgggattagctagttggtgaggtaatggctcaccaaggcgacgatccctagctggtctgagaggatgatcagccacactggaactgagacacggtccagactcctacgggaggcagcagtggggaatattgcacaatgggggaaaccctgatgcagccatgccgcgtgtgtgaagaaggccttcgggttgtaaagcactttcagcgaggaggaaaggttgacgcctaatacgtgtcaactgtgacgttactcgcagaagaagcaccggctaactccgtgccagcagccgcggtaatacggagggtgcaagcgttaatcggaattactgggcgtaaagcgcacgcaggcggttggataagttagatgtgaaagccccgggctcaacctgggaattgcatttaaaactgtccagctagagtcttgtagaggggggtagaattccaggtgtagcggtgaaatgcgtagagatctggaggaataccggtggcgaaggcggccccctggacaaagactgacgctcaggtgcgaaagcgtggggagcaaacaggattagatacctggtagtgcacgccgtaaacgatgtcgatttggaggctgtgtccttgagacgtggcttccggagctaacgcgttaaatcgaccgcctggggagtacggccgcaaggttaaaactcaaatgaattgacgggggcccgcacaagcggtggagcatgtggtttaattcgatgcaacgcgaagaaccttacctggccttgacatgtctggaatcctgcagagatgcgggagtgccttcgggaatcagaacacaggtgctgcatggctgtcgtcagctcgtgtcgtgagatgttgggttaagtcccgcaacgagcgcaacccctgtcctttgttgccagcacgtaatggtgggaactcaagggagactgccggtgataaaccggaggaaggtggggatgacgtcaagtcatcatggcccttacggccagggctacacacgtgctacaatggcgcgtacagagggctgcaagctagcgatagtgagcgaatcccaaaaagcgcgtcgtagtccggatcggagtctgcaactcgactccgtgaagtcggaatcgctagtaatcgcaaatcagaatgttgcggtgaatacgttcccgggccttgtacacaccgcccgtcacaccatgggagtgggttgcaccagaagtagatagcttaaccttcgggagggcgttaccacgg

>16S rDNA sequence of *A. hydrophila* AH-4

gctacacatgcagtcgagcggcagcgggaaagtagcttgctacttttgccggcgagcggcggacgggtgagtaatgcctgggaaattgcccagtcgagggggataacagttggaaacgactgctaataccgcatacgccctacgggggaaagcaggggaccttcgggccttgcgcgattggatatgcccaggtgggattagctagttggtgaggtaatggctcaccaaggcgacgatccctagctggtctgagaggatgatcagccacactggaactgagacacggtccagactcctacgggaggcagcagtggggaatattgcacaatgggggaaaccctgatgcagccatgccgcgtgtgtgaagaaggccttcgggttgtaaagcactttcagcgaggaggaaaggttgatgcctaatacgtatcaactgtgacgttactcgcagaagaagcaccggctaactccgtgccagcagccgcggtaatacggagggtgcaagcgttaatcggaattactgggcgtaaagcgcacgcaggcggttggataagttagatgtgaaagccccgggctcaacctgggaattgcatttaaaactgtccagctagagtcttgtagaggggggtagaattccaggtgtagcggtgaaatgcgtagagatctggaggaataccggtggcgaaggcggccccctggacaaagactgacgctcaggtgcgaaagcgtggggagcaaacaggattagataccctggtagtccacgccgtaaacgatgtcgatttggaggctgtgtccttgagacgtggcttccggagctaacgcgttaaatcgaccgcctggggagtacggccgcaaggttaaaactcaaatgaattgacgggggcccgcacaagcggtggagcatgtggtttaattcgatgcaacgcgaagaaccttacctggccttgacatgtctggaatcctgcagagatgcgggagtgccttcgggaatcagaacacaggtgctgcatggctgtcgtcagctcgtgtcgtgagatgttgggttaagtcccgcaacgagcgcaacccctgtcctttgttgccagcacgtaatggtgggaactcaagggagactgccggtgataaaccggaggaaggtggggatgacgtcaagtcatcatggcccttacggccagggctacacacgtgctacaatggcgcgtacagagggctgcaagctagcgatagtgagcgaatcccaaaaagcgcgtcgtagtccggatcggagtctgcaactcgactccgtgaagtcggaatcgctagtaatcgcaaatcagaatgttgcggtgaatacgttcccgggccttgtacacaccgcccgtcacaccatgggagtgggttgcaccagaagtagatagcttaaccttcgggagggcgttacc

Table S1 Amplification composition and condition of SOE-PCR

| Composition of SOE-PCR | Volume |
| --- | --- |
| Template-*P_slpA_* (0.1 ng/μL) | 1 μL |
| Template-*NHM* (0.1 ng/μL) | 1 μL |
| *P_slpA_*-F | 1 μL |
| *NlpC*-R | 1 μL |
| 5*buffer | 10 μL |
| dNTPs | 4 μL |
| FastPfu DNA ploymerase | 1 μL |
| ddH_2_O | 31 μL |
| Total volume | 50 μL |


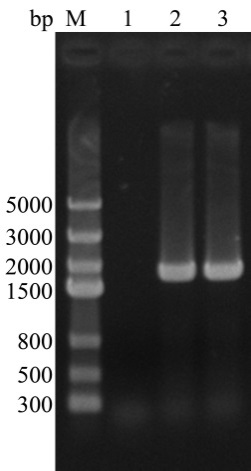


Fig. S1 SOE-PCR product *P_slyA_*-*NHM* gene (1738 bp). M stands for DNA Marker Ⅲ, line 1 stands for the control check, and line 2 stands for SOE-PCR product

Fig. S2 AHL lactonase activity (column chart) and live cells of *Lc*AiiK (line chart) after storage in 10 mM PBS at 4 °C. Data are shown as mean ± standard deviation (sd), n= 3
